# Peri-implantitis in patients without regular supportive therapy: Prevalence and risk indicators

**DOI:** 10.1007/s00784-024-05673-8

**Published:** 2024-04-26

**Authors:** Víctor Ruiz-Romero, Rui Figueiredo, Jorge Toledano-Serrabona, Yehia Abdelazim, Octavi Camps-Font, Yamil Salazar-Salazar, Aina Plana-Soler, Carles Subirà-Pifarré, Eduard Valmaseda-Castellón

**Affiliations:** 1 Oral Surgery and Implantology, Faculty of Medicine and Health Sciences of the University of Barcelona, Barcelona, Spain; 2 Dental and Maxillofacial Pathology and Therapeutics Research Group, IDIBELL Research Institute, Barcelona, Spain; 3 Master of Adult Dental Comprehensive Dentistry, Faculty of Medicine and Health Sciences of the University of Barcelona, Barcelona, Spain

**Keywords:** Peri-implantitis, Dental implants, Prevalence, Risk factors, Gingiva, Dental prosthesis

## Abstract

**Objectives:**

To determine the prevalence of peri-implant diseases in patients treated in a university setting without a regular peri-implant supportive therapy schedule, and to identify the risk indicators associated with peri-implantitis.

**Material and methods:**

A retrospective cohort study was made of patients with dental implants with at least 12 months of functional loading who did not receive regular peri-implant supportive therapy. Patient- and implant-related variables were retrieved, and clinical and radiological examinations were performed. Descriptive and bivariate analyses and multilevel logistic regression analyses were performed to identify factors associated with peri-implantitis.

**Results:**

A total of 213 implants in 88 patients were analyzed. The patient-level prevalence of peri-implantitis and peri-implant mucositis was 26.1% (95%CI: 16.7%—35.5%) and 44.3% (95%CI: 34.0%—54.6%), respectively. Peri-implant diseases were significantly more frequent when the width of the keratinized mucosa was < 2 mm (OR = 5.26; 95%CI: 1.24—22.26; *p* = 0.024), and when there was 12 month post-loading bone loss (OR = 2.96; 95%CI: 1.35—6.52; *p* = 0.007).

**Conclusions:**

Peri-implantitis is a common finding in patients without regular peri-implant supportive therapy (prevalence 16.7–35.5%). A thin peri-implant keratinized mucosa (< 2 mm) and a higher degree of bone remodeling after loading seem to be the main risk factors for peri-implantitis in this patient profile.

**Clinical relevance:**

Patients who do not engage in supportive peri-implant maintenance have a higher risk of peri-implantitis. A thin keratinized mucosa and bone loss during the first year of loading are predisposing factors for peri-implantitis.

## Introduction

Dental implants are a reliable treatment option for replacing missing teeth in edentulous patients [1]. However, several complications may arise after implant placement. Among these, biological complications are found to be the main cause of long-term implant failure [2–4].

Peri-implant mucositis involves inflammation of the peri-implant mucosa (i.e., bleeding on probing, suppuration, erythema, etc.) without bone loss [4]. This complication should be treated as soon as possible to prevent it from progressing to peri-implantitis [5, 6]. In addition to signs of inflammation, this latter condition is characterized by progressive bone loss that compromises dental implant survival [7]. Nonetheless, the exact mechanism of progression from peri-implant mucositis to peri-implantitis remains unclear [8–10].

In the latest consensus report of the European Federation of Periodontology (EFP) and the American Academy of Periodontology (AAP), oral biofilm was identified as the etiological factor responsible for peri-implant disease [11]. Regarding risk factors, patients with a history of periodontitis, as well as those with poor adherence to peri-implant supportive therapy, were found to have a higher risk of peri-implantitis [6–9]. On the other hand, some authors have identified several predisposing/triggering factors that could lead to oral biofilm accumulation and thus increase the risk of developing peri-implantitis [11, 12]. Some examples are a lack of peri-implant keratinized mucosa, an inadequate prosthetic design, the presence of systemic disease conditions, implants placed in augmented sites, and toxic habits [7, 10, 13–17].

Although peri-implant supportive therapy programs are essential to prevent peri-implant diseases, the majority of patients with dental implants (around 60%) fail to attend regular maintenance appointments [18]. The available scientific evidence regarding risk factors/indicators of peri-implantitis in such populations is still scarce. Thus, the aim of the present study was to assess the prevalence of peri-implant diseases in patients with dental implants treated in a university setting who did not attend regular peri-implant supportive therapy, and to determine the risk indicators associated with peri-implantitis in patients with this profile.

## Material and methods

A retrospective cohort study was carried out in the Dental Hospital of the University of Barcelona (Barcelona, Spain). The protocol was approved by the local Ethics Committee (protocol number 21/17), and the study was conducted in accordance with the Declaration of Helsinki [19]. The manuscript followed the Strengthening the Reporting of Observational studies in Epidemiology (STROBE) guidelines [20].

### Study sample

The main inclusion criteria were consecutive patients ≥ 18 years of age that had Avinent® dental implants (Avinent Dental System®, Santpedor, Spain) placed between 2012 and 2021. A minimum of 12 months of functional loading was required. Patients who attended at least two peri-implant supportive therapy appointments per year were excluded. Other exclusion criteria were pregnant patients, patients with systemic conditions that hindered clinical examination and cooperation, and implants without functional loading or with a cemented prosthesis. All patients were recalled and underwent a full clinical and radiological peri-implant examination during 2022 and 2023.

### Case definition

The diagnosis of peri-implant health, peri-implant mucositis and peri-implantitis was established in accordance with the latest consensus report of the World Workshop on the Classification of Periodontal and Peri-Implant Diseases and Conditions [4]:

- Peri-implant health: absence of inflammatory signs, absence of bleeding and/or suppuration on gentle probing (BoP/SoP), no increase in probing depth (PD) compared to previous examinations, and absence of bone loss beyond changes in crestal bone levels resulting from initial bone remodeling.
- Peri-implant mucositis: presence of BoP/SoP with or without an increase in PD compared to previous explorations, and absence of bone loss beyond changes in crestal bone levels resulting from initial bone remodeling.
- Peri-implantitis: presence of BoP/SoP, increased PD compared to previous examinations, and presence of bone loss beyond changes in crestal bone levels resulting from initial bone remodeling. In cases with no available previous records: presence of BoP/SoP, probing depth ≥ 6 mm and bone level ≥ 3 mm apical to the most coronal part of the intraosseous component of the implant.

### Clinical and radiographic assessment

The following patient-related variables were recorded: age, gender, smoking habit, previous history of periodontitis, and systemic diseases. Furthermore, the following clinical parameters were registered:

- Plaque index according to the Loe & Silness index [21].
- BoP scored as positive if bleeding was present during gentle probing.
- SoP scored as positive if pus was present during probing.
- Peri-implant probing pocket depth recorded in millimeters from the tip of the probe to the implant platform.
- Keratinized mucosa (KM) width measured from the free mucosal margin to the mucogingival junction at the mid-buccal, -mesial and -distal line angles.
- Prosthetic emergence profile calculated as the angle between the long axis of the implant and a line tangential to the restoration [22].
- Implant position and characteristics (diameter, length, connection, abutment and implant type).
- Hard or soft tissue augmentation procedures.
- Type of prosthesis (implant-supported crown (ISC), fixed partial dentures (FPD), fixed complete dentures (FCD) or overdentures (OD)).

All prostheses were removed during the peri-implant examination in order to improve the accuracy of the measurements.

In addition, a radiographic evaluation was made using digital periapical radiographs and XCP (extension cone paralleling) positioning devices. Radiographic marginal bone loss was recorded on periapical radiographs using ImageJ® software, measuring the interproximal distance in millimeters from the post-loading bone level (12 ± 3 months after prosthetic loading) to the current bone level. Marginal bone was recorded with a negative value (-) if bone was above the implant platform; with a value of 0 if bone was at the same level as the implant platform; and with a positive value ( +) if the marginal bone was below the implant platform.

### Sample size

The variable smoking was used to calculate the sample size. The risk of developing peri-implantitis in smokers is twice that in non-smokers [23]. Thus, the sample size was found to be 90 patients (alpha = 0.05; statistical power 90%). Patients were considered smokers when they smoked at least 1 cigarette/day as previously reported [24, 25].

### Statistical analysis

The data obtained were entered on a Microsoft Excel spreadsheet (Microsoft®, Redmond, WA, USA) and subsequently processed with the SPSS software version 29 (SPSS Inc., Chicago, IL, USA).

The patient- and implant-level prevalences of peri-implant status (peri-implant health, peri-implant mucositis, peri-implantitis) and corresponding 95% confidence intervals (95%CIs) were calculated.

At patient-level, simple binary logistic regression models were performed to explore the homogeneity of the study groups and scale and categorical variables. At the implant-level, univariate binary logistic regression models using generalized estimating equations (GEE) were performed to identify the association between each covariate with disease recurrence. The GEE method was used to take into account the fact that a single patient may have more than one implant. Crude odds ratios (OR) with their respective 95% confidence intervals (95%CI) were calculated for each covariable.

A multivariate analysis was performed using a GEE binary logistic regression model with a forced entry method to evaluate the effect of the factors that were univariately significant (*P* < 0.1). Adjusted OR including 95% CIs were obtained from the Wald chi-square statistic setting the level of significance at *P* < 0.05. The assumptions underlying the statistical analysis were checked.

## Results

### Demographic data

**Fig. 1:**
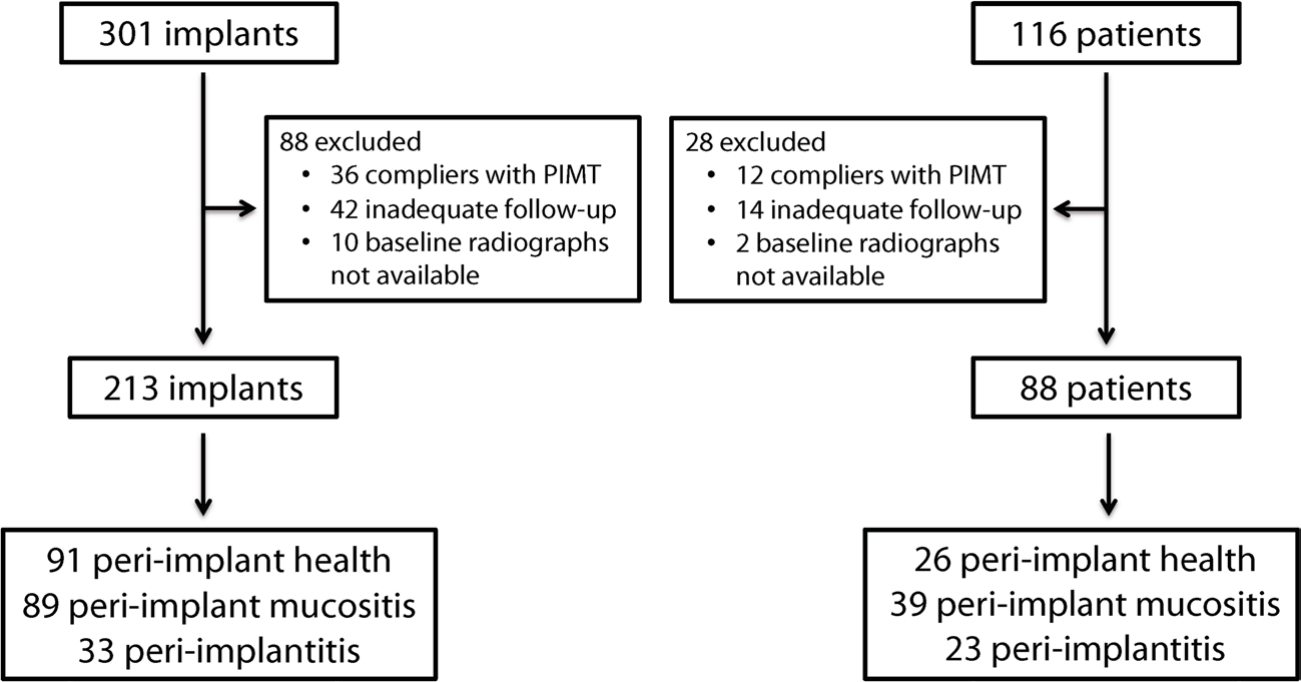
Flow-chart diagram of the participants of the present study. PIMT: Peri-implant maintenance therapy.

**Fig. 2:**
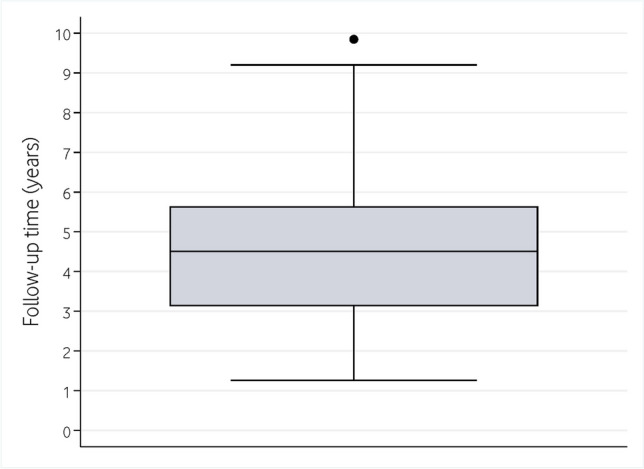
Boxplot illustrating the follow-up time (in years) of the study sample

Out of the 116 enrolled patients (301 implants), 12 (36 implants) were excluded due to their compliance with the peri-implant supportive therapy, and an additional 14 patients (42 implants) were excluded due to insufficient follow-up period after prosthetic loading. Among remaining 90 patients (223 implants), two patients with 10 implants were excluded because baseline radiographs were not available (Fig. 1). Thus, the retrospective analysis included a total of 88 patients (213 implants), with a mean age of 58.7 years (Range: 32 to 82) and a mean follow-up of 4.8 years (standard deviation [SD] = 2.0 years; Range: 1.3 to 9.8) (Fig. 2). The mean annual number of visits for peri-implant maintenance therapy per patient was 0.36 (SD = 0.35). Twenty-three patients did not undergo any maintenance treatment, and 27 patients were only visited once during the follow-up period. Table 1 shows the main implant- and patient-related variables.

**Table 1:** Implant- and patient-related variables

**Patient-variables** (*n* = 88)	**Number (%)**				**Univariate analysis (p)**
	**Health**	**Mucositis**	**Peri-implantitis**	**Total**	
Female	22 (42.3%)	20 (38.5%)	10 (19.2%)	52 (59.1%)	0.134
Smoker	9 (47.4%)	9 (47.4%)	1 (5.2%)	19 (21.6%)	0.786
History of periodontitis	16 (43.2%)	10 (27.1%)	11 (29.7%)	37 (42%)	0.365
Diabetes mellitus	7 (58.3%)	2 (16.7%)	3 (25.0%)	12 (13.6%)	0.064
Need for GBR	6 (50.0%)	4 (33.3%)	2 (16.7%)	12 (13.6%)	0.265
**Implant-variables** (*n* = 213)	**Health**	**Mucositis**	**Peri-implantitis**	**Total**	**Univariate analysis (p)**
Connection design					
External	31 (45.6%)	22 (32.4%)	15 (22.0%)	68 (31.9%)	0.318
Internal	53 (41.7%)	59 (46.5%)	15 (11.8%)	127 (59.6%)	
Conical	7 (38.9%)	8 (44.4%)	3 (16.7%)	18 (8.5%)	
Keratinized mucosa					
< 2 mm	9 (26.5%)	13 (38.2%)	12 (35.3%)	34 (16%)	0.008
≥ 2 mm	82 (45.8%)	76 (42.5%)	21 (11.7%)	179 (84%)	
Type of prosthesis					
Single-crown	51 (48.1%)	43 (40.6%)	12 (11.3%)	106 (49.8%)	0.339
Bridge	32 (39.0%)	33 (40.3%)	17 (20.7%)	82 (38.5%)	
Overdenture	8 (32.0%)	13 (52.0%)	4 (16.0%)	25 (11.7%)	
Prosthesis emergence					
< 30º	88 (43.8%)	86 (42.8%)	27 (13.4%)	201 (94.4%)	< 0.001
> 30º	3 (25.0%)	3 (25.0%)	6 (50.0%)	12 (5.6%)	
Intermediate abutment					
Without abutment	59 (47.6%)	42 (33.9%)	23 (18.5%)	124 (58.2%)	0.304
With abutment	32 (35.9%)	47 (52.8%)	10 (11.3%)	89 (41.8%)	
**Implant-variables** (*n* = 213)	**Mean (SD)**				**Univariate analysis (p)**
	**Health**	**Mucositis**	**Peri-implantitis**	**Total**	
Baseline bone level, mm^†^	0.43 (0.69)	0.41 (0.67)	1.16 (1.09)	0.53 (0.64)	< 0.001
Final bone level, mm	1.02 (0.77)	1.22 (0.76)	2.77 (1.52)	1.37 (1.10)	

*GBR* guided bone regeneration, *SD* standard deviation

^†^12 months post-prosthetic loading

### Implant characteristics

A total of 213 implants with the same surface treatment (Avinent Implant System®, Santpedor, Spain) were analyzed. The following three implant connections were used: internal hexagonal (*n* = 127; 59.6%), external hexagonal (*n* = 68; 31.9%) and internal conical (*n* = 18; 8.5%) (Table 1). Most of the implants were of standard diameter (i.e., > 3.5 mm) (*n* = 186; 86.9%) and length (i.e., ≥ 8 mm) (*n* = 206; 96.7%). The types of prosthesis were ISC in 49.77% (106 implants) of the implants, 38.50% (82 implants) were FPD, and 11.74% (25 implants) were OD. A total of 12 implants (13.6%) were placed after previous hard or soft tissue augmentation procedures.

### Peri-implant diseases

The mean baseline marginal bone level was 0.53 mm (SD = 0.64 mm). The implant- and patient-based prevalence of peri-implant diseases is shown in Table 2. Dental biofilm and BoP were present in 98.6% and 56.81% of the implants, respectively. Only four implants presented SoP.

**Table 2:** Prevalence of peri-implantitis at implant- and patient-based level

	**Implant-based prevalence**			**Patient-based prevalence**		
	*n*	%	95%CI	*n*	%	95%CI
**Peri-implant diagnosis**						
Peri-implant health	91	42.7	32.1 – 53.3	26	29.5	19.3 to 39.7
Peri-implant mucositis	89	41.8	31.3 – 52.3	39	44.3	34.0 to 54.6
Peri-implantitis	33	15.5	10.2 – 20.8	23	26.1	16.7 to 35.5

*CI* confidence interval

### Peri-implantitis indicators

Diabetes mellitus, prosthesis emergence angle, KM and baseline marginal bone loss were associated with peri-implantitis in the bivariate analysis (*p* < 0.10) (Table 1).The multivariate multilevel GEE showed implants with KM width < 2 mm (OR_a_ = 5.26; 95% CI: 1.24 to 22.26; *P* = 0.024) and higher degree of initial physiological bone remodeling (OR_a_ = 2.96; 95%CI: 1.35 to 6.52; *P* = 0.007) (Table 3) to be more susceptible to peri-implantitis (Table 3).

**Table 3:** Multilevel logistic regression analysis based on the generalized estimation equations model

	OR	95%CI	*p*-value
Diabetes mellitus			
No	1		
Yes	1.39	0.22 to 8.95	0.726
Prosthesis emergence angle			
< 30º	1		
> 30º	5.91	0.76 to 46.09	0.090
Keratinized tissue			
≥ 2 mm	1		
< 2 mm	5.26	1.24 to 22.26	0.024*
Baseline MBL	2.97	1.35 to 6.52	0.007*

*MBL* marginal bone loss, *CI* confidence interval, *OR* odds ratio

^*^Statistically significant (*p* < 0.05)

## Discussion

Peri-implantitis is a multifactorial biological complication induced by biofilm accumulation [2, 26]. Thus, peri-implant supportive therapy plays a crucial role in disease prevention [9, 11]. The present study, having only included patients who did not follow a regular peri-implant maintenance program, seems to support this statement, since peri-implantitis was common (present in approximately 1 out of 4 patients). In fact, peri-implantitis risk was higher than reported by Rakic et al. [27] (18.5%) and Derks and Tomasi [28] (22%). Besides, a previous cross-sectional study by our research group found a lower rate of peri-implantitis (16.3%) in patients who followed a periodontal maintenance program [29]. Supporting evidence from other studies further emphasizes the significance of compliance with peri-implant therapy in reducing peri-implantitis risk. For instance, Monje et al. [30] demonstrated a lower prevalence (4.5%) in compliant patients compared to their non-compliant counterparts (26.3%), and Costa et al. [31] reported a prevalence of peri-implantitis in regular patients of 18% vs. 43.9% in non-compliant patients. Therefore, the high prevalence found in the present sample is probably related to the lack of adequate peri-implant maintenance therapy, aligning with recent studies addressing the impact of compliance on peri-implant outcomes [32, 33]. Hence, it is the current authors’ desire to call for future controlled longitudinal studies to assess the impact of peri-implant maintenance therapy on peri-implant diseases. This comparative investigation could provide valuable insights into the influence of patient compliance on the long-term outcomes and management of peri-implant health. Additionally, various factors such as the frequency and content of maintenance visits may contribute to a more comprehensive understanding of effective strategies for preventing and managing peri-implant diseases.

This paper has some limitations that need to be considered. Firstly, the retrospective nature of the study does not allow us to establish a direct cause-effect relationship between the abovementioned risk indicators and peri-implantitis. Also, data retrieval could be compromised because of inconsistent recording or memory biases. However, since all patients were recalled for a clinical and radiological examination, the final peri-implant diagnosis is likely to be accurate. Another drawback of the present study is related to its small sample size, which resulted in broad confidence intervals. Furthermore, the assumption regarding tobacco use, which was made to anticipate the required sample size, was not met. In order to correct this fact, a post-hoc power analysis was performed. In this sense, a sample size of 213 independent implants provided 91% power at confidence 95% to detect an OR of 3 using a logistic regression model. However, due to the multi-level design of the data (each patient provided an average of 2.4 implants), the power was corrected assuming a moderate intra-subject correlation (ρ = 0.5) resulting in a power of 73%.

The present outcomes seem to underline the importance of KM in the risk of developing peri-implantitis in patients without a regular implant supportive therapy schedule. This association might be related to the increased difficulty and discomfort of performing adequate hygiene in these cases. Unattached mucosa might enhance the penetration of bacterial biofilm into the peri-implant sulcus or render its removal more difficult [12]. Moreover, a study that included patients that underwent less than two maintenance visits per year found that an amount of KM width < 2 mm appeared to be associated with higher peri-implantitis risk [18]. Ramanauskaite et al. [34] also concluded that insufficient KM width was linked to an increased prevalence of peri-implantitis. Furthermore, the presence of KM seems to have a positive influence upon immunological features [12]. However, a retrospective study analyzing the effect of KM on peri-implant health concluded that KM width had no effect upon the prevalence of peri-implant diseases [35]. This is in contrast with our own results, the difference being that in the previous report all patients were following a strict maintenance regimen. Indeed, in non-compliant patients, KM width might be particularly relevant.

Marginal bone loss (MBL) is a fundamental factor for the development of peri-implantitis and has been considered a key criterion for assessing implant success. In general, a physiological bone remodeling process is considered normal during the first year after loading, and an annual bone loss of < 0.2 mm might be acceptable after the initial 12 months [36]. However, recent research has suggested that MBL > 0.50 mm in the first 6 months after prosthetic loading is a risk indicator for peri-implant bone loss progression [37, 38]. In our study, the mean baseline MBL in the first year was above this threshold (0.53 mm). If such bone remodeling leads to exposure of the implant surface, or mucosal recession leaves open space in the interproximal areas, extensive biofilm growth is likely to appear in just a few days [39]. Consequently, peri-implant tissue inflammation might provoke faster and more pronounced progressive bone loss. The outcomes of the present study seem to support this theory, since the bone level after 12 months of loading was significantly associated to the development of peri-implantitis.

In the present study, no significant relationship was found between implants placed in pristine and augmented sites regarding the incidence of peri-implant biological complications. This observation aligns with recent publications demonstrating no statistically significant differences in the patient-based prevalence of peri-implantitis between implants placed in pristine sites (10.3%; 95% CI: 4 to 17%) and augmented sites (17.8%; 95% CI: 0 to 37%) [40].

The high prevalence of peri-implantitis observed over a short follow-up period may also be related to the implant surface characteristics. This perspective is consistent with existing literature that emphasizes the correlation between implant surface roughness and the occurrence of peri-implantitis [41, 42]. Conversely, a recent meta-analysis showed no differences between the different surfaces during the onset of peri-implantitis [43]. Nonetheless, this factor remained beyond the scope of evaluation in our study, as all implants featured an identical moderately rough microdesign (i.e., Avinent® Biomimetic obtained through a combination of shot blasting and electrochemical treatment with a Ca- and *P*-rich electrolyte solution).

Systemic conditions could also favor peri-implant diseases. In diabetic patients, bleeding on probing and bone loss around the implant seem to increase with blood glucose metabolic decompensation [44]. In the present study, diabetic patients also seemed to have a slightly higher likelihood (*p* = 0.064 in the univariate analysis) of developing peri-implantitis, though the difference was not statistically significant (Table 1).

It has been proposed that peri-implantitis follows a non-linear, accelerating pattern, with the majority of cases initiating within the first 3 years of implant function [45]. The findings reported by Derks et al. [45] are in agreement with results presented in this study,  as patients with longer follow-up (i.e., > 3 years) were almost 5 times more likely to exhibit peri-implantitis (OR = 4.83; IC95%: 1.04 to 22.17; *p* = 0.044). This observation implies a potentially aggravated scenario in the near future. Consequently, future research with longer observational periods is needed to confirm this association.

Several authors have pointed out that the prosthetic design must allow correct access for oral hygiene, in order to prevent biological complications [12, 46]. A wide emergence angle and a convex profile could increase the risk of peri-implantitis, since these factors may hamper biofilm removal [47]. Indeed, Katafuchi et al. [17] found an emergence angle of > 30º to be a significant risk indicator. Furthermore, prosthetic adjustment was suggested to be an important step in the peri-implant mucositis treatment in conjunction with anti-infective nonsurgical management [47]. Our results seem to support this statement, since peri-implant disease was associated to an increased emergence angle in the bivariate analysis (Table 1). Interestingly, the greater the amount of initial bone loss, the greater the probability of presenting a prosthesis with an angle > 30° (OR = 1.50; 95% CI: 1.02 to 2.21; *p* = 0.048). Accordingly, future studies should evaluate whether the amount of bone loss during the osseointegration period determines the emergence angle of the prosthesis.

Regarding the type of prosthesis, no differences were found between the prosthetic approach adopted. Although fixed prostheses are usually associated with a higher risk of developing peri-implantitis possibly due to the difficulty of access for hygiene [46], in our sample, patients rehabilitated with overdentures also presented high rates of peri-implant diseases (Risk = 16%; 95%CI: 6.4 to 34.7). This finding could be partially explained by the fact that not attending regular maintenance visits is more crucial than the specific type of prosthetic restoration per se. Additionally, it is important to consider that patients with overdentures have lost all their teeth, potentially leading to diminished attention to oral hygiene. This, in turn, appears to influence the compliance risk profile [40].

## Conclusions

Peri-implantitis is a common finding in patients without a regular peri-implant supportive therapy schedule, with a prevalence that ranges from 16.7–35.5%. Less than 2 mm of KM and a higher degree of bone remodeling after one year of loading seem to be the main risk factors for peri-implantitis in patients who do not regularly follow implant maintenance.

## Data Availability

The data supporting the findings of this study are available on reasonable request from the corresponding author.
